# Risk Factors for Prolonged Mechanical Ventilation After Pulmonary Endarterectomy: 7 Years' Experience From an Experienced Hospital in China

**DOI:** 10.3389/fsurg.2021.679273

**Published:** 2021-06-10

**Authors:** Congya Zhang, Lijing Yang, Sheng Shi, Zhongrong Fang, Jun Li, Guyan Wang

**Affiliations:** ^1^Department of Anesthesiology, Beijing Tongren Hospital, Capital Medical University, Beijing, China; ^2^Department of Anesthesiology, National Center of Cardiovascular Diseases, Chinese Academy of Medical Sciences and Peking Union Medical College, Fuwai Hospital, Beijing, China

**Keywords:** prolonged mechanical ventilation, chronic thromboembolic pulmonary hypertension, pulmonary endarterectomy, risk factors, outcome

## Abstract

**Background:** Prolonged mechanical ventilation (PMV) is common after cardiothoracic surgery, whereas the mechanical ventilation strategy after pulmonary endarterectomy (PEA) has not yet been reported. We aim to identify the incidence and risk factors for PMV and the relationship between PMV and short-term outcomes.

**Methods:** We studied a retrospective cohort of 171 who undergoing PEA surgery from 2014 to 2020. Cox regression with restricted cubic splines was performed to identify the cutoff value for PMV. The Least absolute shrinkage and selection operator regression and logistic regressions were applied to identify risk factors for PMV. The impacts of PMV on the short-term outcomes were evaluated.

**Results:** PMV was defined as the duration of mechanical ventilation exceeding 48 h. Independent risk factors for PMV included female sex (OR 2.911; 95% CI 1.303–6.501; *P* = 0.009), prolonged deep hypothermic circulatory arrest (DHCA) time (OR 1.027; 95% CI 1.002–1.053; *P* = 0.036), increased postoperative blood product use (OR 3.542; 95% CI 1.203–10.423; *P* = 0.022), elevated postoperative total bilirubin levels (OR 1.021; 95% CI 1.007–1.034; *P* = 0.002), increased preoperative pulmonary artery pressure (PAP) (OR 1.031; 95% CI 1.014–1.048; *P* < 0.001) and elongated postoperative right ventricular anteroposterior dimension (RVAD) (OR 1.119; 95% CI 1.026–1.221; *P* = 0.011). Patients with PMV had longer intensive care unit stays, higher incidences of postoperative complications, and higher in-hospital medical expenses.

**Conclusions:** Female sex, prolonged DHCA time, increased postoperative blood product use, elevated postoperative total bilirubin levels, increased preoperative PAP, and elongated postoperative RVAD were independent risk factors for PMV. Identification of risk factors associated with PMV in patients undergoing PEA may facilitate timely diagnosis and re-intervention for some of these modifiable factors to decrease ventilation time and improve patient outcomes.

## Introduction

Chronic thromboembolic pulmonary hypertension (CTEPH) is the main cause of type IV pulmonary hypertension (PH), which is defined as a mean pulmonary arterial pressure (mPAP) >25 mmHg with a pulmonary capillary wedge pressure (PCWP) <15 mmHg (1–3). The major pathological mechanism is elevated pulmonary vascular resistance (PVR) due to the obstruction of pulmonary embolism and incomplete thrombus resolution within the pulmonary arteries, which eventually contribute to progressive PH and right ventricular failure (4). The incidence of CTEPH in the general population and among acute pulmonary embolism survivors is 3–30 cases per million and 0.4–9.1%, respectively (2). Without appropriate treatment, the 3-year survival rate of patients with an mPAP >50 mmHg is not >10% (5). Pulmonary endarterectomy (PEA) remains the predominant guideline-recommended treatment for patients with CTEPH (6). The short-term and long-term outcomes of PEA are reasonable in experienced centers (7, 8), with in-hospital mortality not >5% (9).

Prolonged mechanical ventilation (PMV) after cardiothoracic surgery is associated with increased mortality and morbidity (10–14). For complex surgical procedures, such as cardiothoracic surgeries, the duration of postoperative mechanical ventilation is generally prolonged (14–16). The reported incidence of PMV after cardiothoracic surgeries varies from 2 (17) to 43 (18), according to different definitions of PMV and different patient selection with different surgery types. Numerous studies examined the independent risk factors for postoperative PMV in various cardiothoracic surgeries to identify modifiable factors (17, 19–21). Early identification and early prevention are central efforts to improving the prognosis of patients.

Due to the characteristics of the PEA procedure, including a longer cardiopulmonary bypass (CPB) time with deep hypothermic circulatory arrest (DHCA) to achieve a bloodless visual field and direct intraoperative stretching of the pulmonary artery, the incidence of postoperative pulmonary complications is much higher than other cardiothoracic surgeries. The major complications after PEA are reperfusion pulmonary edema and pulmonary infection, which are not beneficial to the prognosis of patients (22). Therefore, the immediate identification of patients with a higher risk profile and the implementation of positive preventive and therapeutic strategies targeting pulmonary complications after PEA is particularly important. The incidence of PMV after PEA was not reported in previous studies. Therefore, the present study identified the incidence and potential risk factors for PMV after PEA and to clarify the relationship between the PMV and short-term outcomes.

## Materials and Methods

### Study Population

From January 2014 to August 2020, patients undergoing PEA with CPB at Fuwai Hospital were consecutively included in this retrospective observational study. We excluded three patients who were not finally diagnosed with CTEPH at discharge and two patients who had incomplete clinical data. The final study population was 171 patients. All patients were given general anesthesia and postoperative targeted management according to the clinical practice in our hospital. The Research Ethics Committees of Fuwai Hospital approved this study.

### Data Collection and Variable Definitions

All data were collected from the electronic medical record system, and two independent researchers were responsible for checking the data quality. Potential predictor variables were chosen based on the previous literature review, clinical judgment, and availability in the authors' hospital. Perioperative characteristics included age, sex, body mass index (BMI), blood type, University of California San Diego (UCSD) classification, New York Heart Association (NHYA) heart function classification, arterial partial pressure of oxygen (PaO_2_) and arterial partial pressure of carbon dioxide (PaCO_2_) inhaling room air on admission to the hospital, deep venous thrombosis history, acute pulmonary embolism history, acute thrombolysis history, antiphospholipid syndrome history, diabetes history, hyperlipidemia history, hypertension history, coronary artery disease (CAD) history, smoking history, concomitant cardiac operation, intraoperative sufentanil use, intraoperative etomidate use, intraoperative midazolam use, intraoperative dexmedetomidine use, intraoperative blood product use, intraoperative total amount of fluid, intraoperative fibrinogen use, intraoperative prothrombin use, intraoperative methylprednisolone use, intraoperative creatine phosphate sodium use, intraoperative ulinastatin use, intraoperative maximum vasoactive-inotropic score (VISmax), CPB time, aortic cross-clamp time, DHCA time, lowest rectal temperature during CPB, lowest nasopharyngeal temperature during CPB, intraoperative blood loss, postoperative blood product use within the first 72 h, and chest discharge within 72 h after surgery. The following preoperative and postoperative biomarkers were collected: white blood cell, monocyte, lymphocyte, neutrophil, hematocrit, platelet counts and hemoglobin levels; international normalized ratio (INR), prothrombin time (PT), activated partial thromboplastin time (APTT), erythrocyte sedimentation rate (ESR), total protein, albumin, alkaline phosphatase, aspartate aminotransferase, alanine aminotransferase, total bilirubin, serum creatinine, blood glucose, lactate dehydrogenase, creatine kinase and isoenzyme of creatine kinase-MB, and C-reactive protein (CRP) levels. The following preoperative and postoperative echocardiography and right heart catheterization variables were collected: left ventricle end-diastolic diameter, left ventricle ejection fraction, atrioventricular valve regurgitation, right ventricle anteroposterior dimension (RVAD), internal diameter of the major pulmonary artery, mPAP, PVR, cardiac output (CO) and cardiac output index (CI).

PMV was defined as the duration of postoperative mechanical ventilation exceeding the cutoff point. According to the results of the Cox regression model for predicting the postoperative length of hospital stay (p-LOHS), the cutoff value for PMV was chosen at which the hazard ratio of prolonged p-LOHS had the highest increasing trend. The VIS was calculated as dopamine (μg/kg/min) + dobutamine (μg/kg/min) + 10 × milrinone (μg/kg/min) + 50 × levosimendan (μg/kg/min) + 100 × epinephrine (μg/kg/min) + 100 × norepinephrine (g/kg/min) + 10,000 × vasopressin (U/kg/min) (23).

### Management in Patients Undergoing PEA Surgery

All patients underwent PEA with CPB. The surgical procedure and anesthesia management of PEA were demonstrated in detail in previous studies (22, 24). Briefly, the key to PEA is completing the bilateral endarterectomy of chronically thromboembolic pulmonary tissue *via* median sternotomy by CPB with intermittent DHCA. A circulatory arrest is inevitable to provide a bloodless surgical field, and deep hypothermia decreases the metabolism and oxygen demand of organs to play a protective role. Each duration of cardiac arrest is within the limit of 20 min, and the maintenance of proper cerebral oxygen saturation and selective cerebral perfusion (SACP) are of great importance to achieve effective cerebral protection.

All patients were transferred to the cardiac intensive care unit (ICU) after PEA. Postoperative extubation was performed when patients met the following standard institution protocols: spontaneous respiration, intact airway reflexes, manageable airway secretions, and hemodynamic stabilization. Postoperative ICU management was provided by a multidisciplinary team of surgeons, radiologists, cardiac anesthesiologists, intensivists, and nurses to provide highly specialized care for CTEPH patients.

### Statistical Analysis

Continuous variables are shown as medians (interquartile ranges), and categorical variables are shown as counts (percentages). We used the non-parametric Mann–Whitney *U*-test to compare continuous variables, and the chi-squared test to compare categorical variables. Multivariate Cox regression with restricted cubic splines was performed to identify the cutoff value for PMV using the p-LOHS as the dependent variable, and the spline was plotted with three knots defined at the 5th, 50th, and 95th percentiles of the variable (25). Least absolute shrinkage and selection operator (LASSO) regression is the most popular and widely used regularized regression which was used to select variables by minimizing the potential collinearity and overfitting of variables with the help of an optimal lambda (λ) (26, 27). The most significant advantage of LASSO regression is that by penalizing all the coefficients of the variables, the coefficients of the relatively insignificant independent variables become zero and were thus excluded from the modeling. LASSO regression can treat all the variables simultaneously, rather than stepwise, which makes the modeling much more stable. LASSO regression is essential when a study has a relatively small sample size but many candidate variables, and it may help the selection of variables that are most important to include in a prediction model. Univariate and multivariate logistic regressions with the stepwise backward elimination method were used to identify risk factors for PMV. The area under the receiver operating characteristic curve (AU-ROC) was used to assess the discrimination of the logistic regression model, and the Hosmer–Lemeshow test was performed to evaluate its calibration. Two-sided *P* < 0.05 was considered significant. All statistical analyses were performed using Stata 14 software and R software (version 3.5.2).

## Results

### Study Population

There were 176 PEA surgeries performed at Fuwai Hospital between January 2014 and August 2020. After excluding three patients who were not finally diagnosed with CTEPH at discharge and two patients who had incomplete clinical data, the final study population contained 171 patients.

### Study Patient Characteristics

Of the 171 patients in this retrospective study, 61 patients (35.7%) were female, with a mean age of 46.79 ± 13.34 years. All patients were diagnosed with CTEPH. Six (3.5%) patients in our population had diabetes mellitus (DM), 28 (16.4%) patients had hypertension, 12 (7%) patients had CAD and 36 (21.1%) patients were smokers. The mean CPB and aortic cross-clamp times in the total study cohort were 224 ± 52 and 111 ± 43 min, respectively. DHCA was performed in all cases with a mean time of 35 ± 16 min. There was no difference in preoperative respiratory function between the PMV group and the non-PMV group.

### Data-Derived Definition of PMV

**Figure 1:**
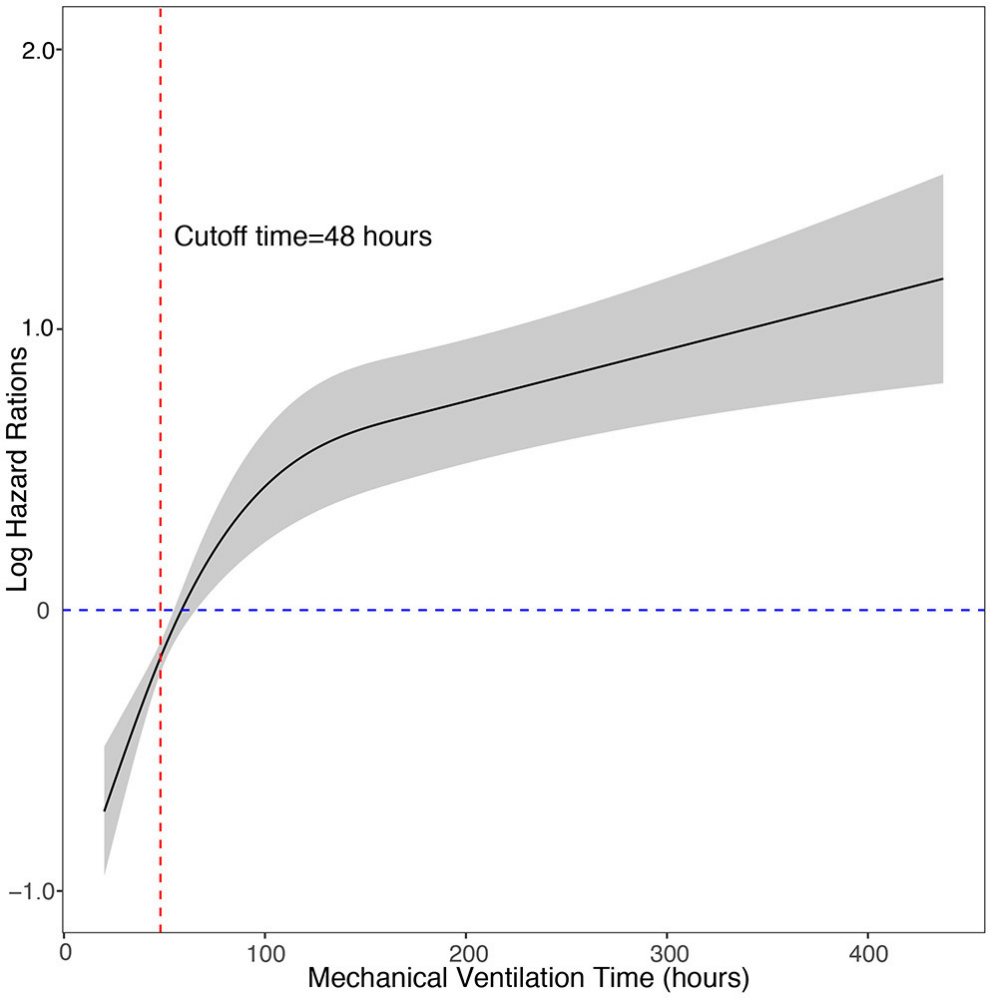
Cox regression model with restricted cubic splines of the relationship between the postoperative mechanical ventilation time and the risk of prolonged length of hospital stay. The log of the adjusted hazard ratios of mechanical ventilation time derived from the multivariate Cox model after adjustment of pulmonary hypertension crisis, pulmonary infection, and pericardial effusion is shown on the y-axis. The 95% confidence intervals of the adjusted hazard ratios are represented by the gray area. The risk function demonstrates an inflection point at 48 h.

We used multivariate Cox regression with the enter method to identify the short-term outcomes that may affect the duration of p-LOHS, and included extracorporeal membrane oxygenation treatment (ECMO), mechanical ventilation time, reintubation, reperfusion pulmonary edema, pulmonary hypertension crisis, pulmonary infection, pericardial effusion, postoperative arrhythmia, renal replacement treatment (RRT), chest drainage within 72 h after surgery, postoperative high-sensitivity C-reactive protein level, postoperative alanine aminotransferase level and postoperative aspartate aminotransferase level. In the Cox regression model, postoperative mechanical ventilation time, pulmonary hypertension crisis, pulmonary infection, and pericardial effusion were independent risk factors for prolonged p-LOHS. Then the restricted cubic spline Cox model was used to analyze the relationship between the mechanical ventilation time and the risk of prolonged p-LOHS. The log of the adjusted hazard ratios of mechanical ventilation time derived from the multivariate Cox model after adjustment of pulmonary hypertension crisis, pulmonary infection, and pericardial effusion was shown on the y-axis, and the mechanical ventilation time was shown on the x-axis. The 95% confidence intervals of the adjusted hazard ratios are represented by the gray area. The cutoff value for PMV was determined to be 48 h because patients with mechanical ventilation time >48 h had sharply increased risks of prolonged p-LOHS (Figure 1). The adjusted hazard ratio of PMV was 1.81 (1.3–2.5).

### Incidence and Risk Factors of PMV

According to the cutoff value we found, the incidence of postoperative PMV in our study group was 50.3% (*n* = 86). The mean ventilation time of the whole cohort was 98 h and the median was 49 h. Table 1 shows the demographic and perioperative variables of the two groups. There were significant differences between the two groups in preoperative cardiac status evaluated using an echocardiogram, preoperative PAP, preoperative PVR, preoperative total bilirubin, CPB time, and intraoperative VISmax.

**Table 1:** Perioperative characteristics of study patients (*n* = 171).

**Variables**	**Non-PMV (*n* = 85)**	**PMV (*n* = 86)**	***p*-value**	**Overall (*n* = 171)**
Age (y)	47 (33, 55)	52 (40, 58)	0.135	50 (34, 57)
Female sex (%)	28 (32.9%)	33 (38.4%)	0.631	61 (35.7%)
Body mass index (Kg/m^2^)	23.7 (21.2, 26.3)	22.45 (20.29, 24.5)	0.013	23.1 (20.9, 25.4)
Diabetes (%)	5 (5.9%)	1 (1.2%)	0.111	6 (3.5%)
Hypertension (%)	14 (16.5%)	14 (16.3%)	0.946	28 (16.4%)
Coronary artery disease (%)	4 (4.7%)	8 (9.3%)	0.370	12 (7%)
Smoking (%)	13 (15.3%)	23 (26.7%)	0.072	36 (21.1%)
PaO_2_ (mmHg)	52.1 (39.1, 65)	51.7 (35, 64.4)	0.273	51.9 (38, 64.8)
PaCO_2_ (mmHg)	38.3 (34.6, 43.5)	38.2 (33.7, 42.5)	0.542	38.2 (34, 43)
FEV1.0 (L)	2.52 (1.97, 3.16)	2.24 (1.69, 2.67)	0.071	2.26 (1.8, 2.95)
FEV1.0 (% of predicted value)	79 (68, 91.5)	73.5 (62.5, 81)	0.055	77 (64.5, 88)
Vital capacity (L)	3.59 (2.68, 4.35)	3.24 (2.33, 3.66)	0.044	3.33 (2.57, 4.01)
Vital capacity (% of predicted value)	86 (79, 94.5)	83.5 (71.5, 89.5)	0.163	84 (73.5, 92.5)
Anteroposterior diameter of right ventricular (mm)	28 (25, 34.5)	34 (29, 41.2)	<0.001	31 (27, 38)
left ventricular end-diastolic diameter (mm)	44 (40, 47)	40 (35.8, 45)	<0.001	43 (38, 46)
Internal diameter of major pulmonary artery (mm)	29 (24, 31.5)	31 (27, 35)	<0.001	30 (25, 33)
Left ventricular ejection fraction (%)	65 (62, 71)	67.1 (60, 73.5)	0.437	66 (60, 72)
NYHA class III	55 (64.7%)	62 (72.1)	0.327	117 (68.4%)
NYHA class IV	5 (5.9%)	1 (1.2%)	0.094	6 (3.5%)
Mean pulmonary arterial pressure in RHC (mmHg)	44 (31.5, 51)	51.5 (46, 59)	<0.001	48 (40, 56.5)
Pulmonary vascular resistance in RHC (Wood U)	7 (5.09, 0.02)	9.52 (7.46, 14.9)	<0.001	8.4 (6.0, 11.7)
Cardiac output in RHC (L min^−1^)	4.73 (3.99, 5.65)	4.43 (3.65, 5.27)	0.129	4.53 (3.76, 5.51)
Preoperative hematocrit (L/L)	0.426 (0.385, 0.459)	0.439 (0.417, 0.470)	0.044	0.432 (0.401, 0.458)
Preoperative alkaline phosphatase (IU/L)	61 (51, 79)	70.5 (55.8, 94.8)	0.016	65 (54, 87)
Preoperative total bilirubin (μmol/L)	13.9 (10.5, 19.3)	21.6 (13.2, 28.9)	<0.001	16.7 (11.5, 24.3)
Preoperative serum creatinine (μmol/L)	79.3 (69.5, 93.2)	87.1 (73.8, 99.9)	0.036	82 (71.1, 95.6)
Preoperative isoenzyme of creatine kinase-MB	9 (2.37, 12)	11 (8, 13)	0.001	10 (7, 13)
**Surgical details**				
Red blood cells use (%)	0 (0%)	2 (2.3%)	0.497	2 (1.2%)
Fresh frozen plasma use (%)	3 (3.5%)	11 (12.8%)	0.048	11 (6.4%)
Platelets use (%)	4 (4.7%)	6 (7%)	0.746	6 (3.5%)
Procedure time (min)	325 (292, 362)	335 (306.5, 375.3)	0.084	330 (302, 370)
Cardiopulmonary bypass time (min)	211 (175.5, 245)	235.5 (214.5, 252.3)	<0.001	233 (189, 250)
Aortic cross-clamp time (min)	98 (79, 120)	112 (95.5, 131)	0.003	106 (85, 124)
DHCA time (min)	32 (21, 43.5)	41 (31, 48.3)	0.001	37 (27, 46)
Lowest rectal temperature (°C)	19.6 (18.7, 20.3)	19.2 (18.6, 20)	0.135	19.3 (18.6, 20.1)
Lowest nasopharyngeal temperature (°C)	17.9 (17.6, 18.3)	18 (17.7, 18.5)	0.455	18 (17.6, 8.4)
Maximal VIS during surgery	8 (3, 14.7)	15 (7, 23)	<0.001	10 (5.0, 18.5)

*PMV, prolonged mechanical ventilation; PaO2, Arterial partial pressure of oxygen; PaCO_2_, Arterial partial pressure of carbon dioxide; FEV1, Forced expiratory volume 1st second; RHC, Right heart catheterization; DHCA, Deep hypothermia circulatory arrest; VIS, maximum vasoactive-inotropic score during surgery*.

**Figure 2:**
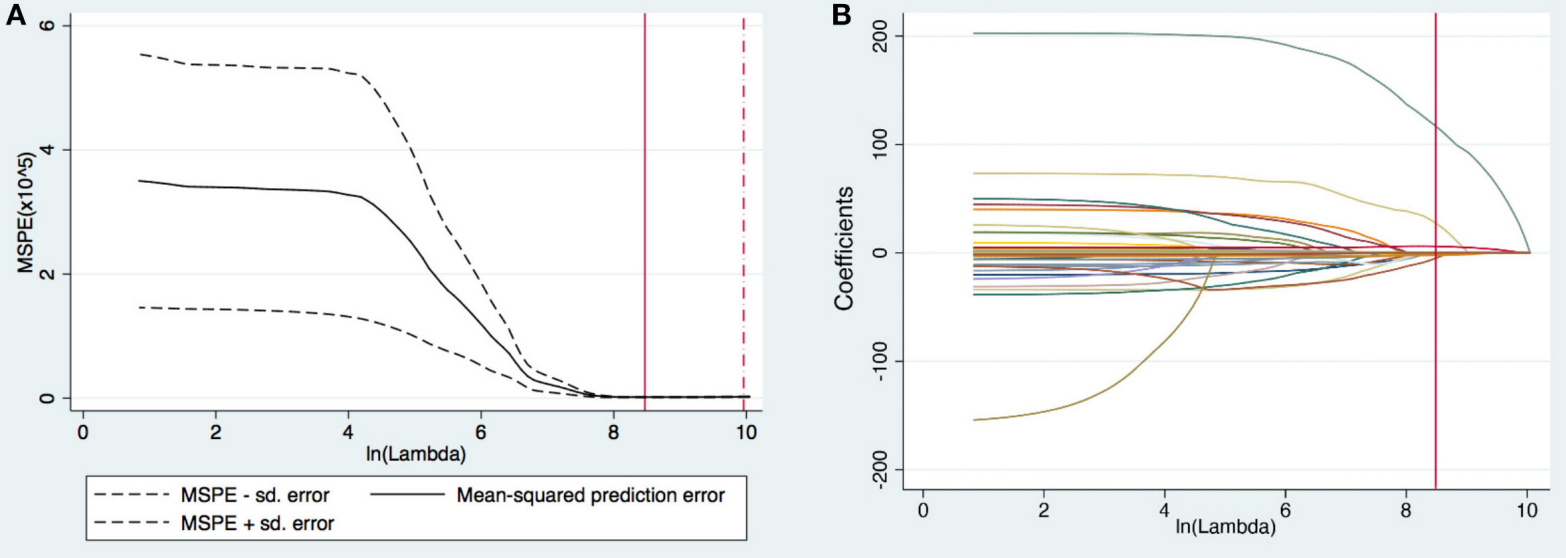
**(A)** Selection of tuning parameter (lambda [λ]) in the least absolute shrinkage and selection operator (LASSO) model via 10-fold cross-validation. The optimal ln (λ) = 8.4703, was chosen based on minimum criteria. **(B)** LASSO coefficients produced by the LASSO logistic regression analysis. Twenty-eight variables (total 102 variables) with non-zero coefficients were chosen in the LASSO logistic regression model based on the optimal ln λ-value and were chosen as the most important variables after considering the correlation among candidate variables.

**Figure 3:**
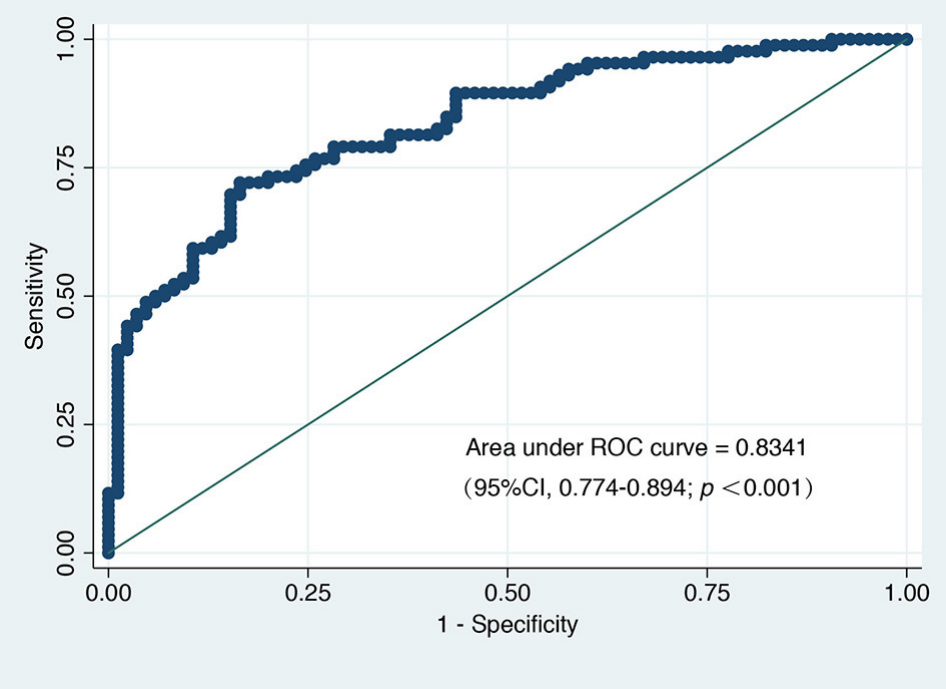
The area under the receiver operating characteristic curve (AU-ROC) for prolonged mechanical ventilation.

A total of 102 variables were included in the LASSO regression, and 28 important variables were chosen at an ln λ of 8.4703. The LASSO regression is shown in Figure 2. Six of these 28 variables, including female sex, prolonged DHCA time, increased postoperative blood product use, elevated postoperative total bilirubin, increased preoperative PAP, and elongated postoperative RVAD were finally included in the logistic regression model and identified as independent risk factors for PMV (Table 2). The appropriateness of the logistic regression model was authorized by the Hosmer–Lemeshow goodness-of-fit statistic (*p* = 0.657). The area under the curve of ROC for PMV was 0.8341 (95% CI, 0.774–0.894) (Figure 3).

**Table 2:** Univariate and multivariate analysis odds ratios for prolonged mechanical ventilation.

**Variables**	**Univariate analysis**		**Multivariate analysis**	
	**OR (95% CI)**	***p*-value**	**OR (95% CI)**	***p*-value**
Female sex	1.268 (0.677–2.373)	0.459	2.911 (1.303–6.501)	0.009
UCSD classification	0.783 (0.305–2.009)	0.223		
Deep venous embolism	1.142 (0.613–2.126)	0.677		
Acute pulmonary embolism	0.920 (0.444–1.905)	0.821		
Hyperlipidemia	0.609 (0.307–1.207)	0.156		
Hypertension	0.986 (0.439–2.217)	0.973		
Smoking	2.022 (0.946–4.321)	0.069		
Intraoperative blood product use	2.167 (0.828–5.671)	0.115		
Intraoperative total amount of fluid (ml)	1.000 (0.999–1.001)	0.772		
Intraoperative etomidate use	0.954 (0.900–1.011)	0.112		
Maximal VIS during surgery	1.041 (1.011–1.071)	0.007		
CPB time (min)	1.014 (1.006–1.022)	<0.001		
DHCA time (min)	1.015 (1.004–1.027)	0.006	1.027 (1.002, 1.053)	0.036
Postoperative blood product use within first 72 h	2.400 (1.135–5.074)	0.022	3.542 (1.203, 10.423)	0.022
Chest drainage within first 72 h (ml)	0.998 (0.997–0.999)	0.006		
Preoperative lymphocyte (*10^9^/L)	1.003 (0.694–1.448)	0.988		
Preoperative platelet (*10^9^/L)	0.999 (0.994–1.003)	0.543		
Preoperative total protein (g/L)	1.002 (0.954–1.054)	0.923		
Preoperative creatine kinase (IU/L)	0.998 (0.991–1.004)	0.471		
Preoperative prothrombin time (s)	0.997 (0.990–1.003)	0.317		
Postoperative aspartate aminotransferase (IU/L)	1.006 (0.998–1.014)	0.121		
Postoperative alkaline phosphatase (IU/L)	1.012 (0.996–1.028)	0.140		
Postoperative isoenzyme of creatine kinase-MB (IU/L)	1.029 (1.008–0.0500)	0.006		
Postoperative total bilirubin (μmol/L)	1.021 (1.009–1.033)	<0.001	1.021 (1.007–1.034)	0.002
Postoperative hemoglobin (g/dL)	0.987 (0.967–1.009)	0.242		
Preoperative left ventricular end diastolic diameter (mm)	0.917 (0.871–0.967)	0.001		
Preoperative pulmonary artery pressure (mmHg)	1.036 (1.022–1.052)	<0.001	1.031 (1.014–1.048)	<0.001
Postoperative anteroposterior diameter of right ventricular (mm)	1.146 (1.065–0.234)	<0.001	1.119 (1.026–1.221)	0.011

*UCSD classification, The University of California, San Diego (UCSD) classification; VIS, maximum vasoactive-inotropic score during surgery; CPB, Cardiopulmonary bypass; DHCA, Deep hypothermia circulatory arrest*.

### Short-Term Outcomes

The 90-day mortality was 1.8% (3/171). There was no 90-day mortality difference (*p* = 0.246) between PMV patients and non-PMV patients, but all three deaths occurred in the PMV group. Of the 171 patients, 86 (50.3%) required PMV. Twelve patients (14%) in the PMV group required tracheotomy, and no patients in the non-PMV group required tracheotomy. Compared to patients without PMV, patients with PMV were associated with more blood transfusions (30.2 vs. 15.3%, *p* = 0.028), a higher rate of delirium (17.4 vs. 5.9%, *p* = 0.011), reperfusion pulmonary edema (9.3 vs. 1.2%, *p* = 0.047), pulmonary infection (51.2 vs. 24.7%, *p* < 0.001) and RRT (7 vs. 0%, *p* = 0.029), prolonged length of ICU stay (LOIS) (4 (3–5) days vs. 6 (5–10) days, *p* < 0.001), and increased in-hospital medical expenses (22.15 [18.59–25.89] $1,000 vs. 27.38 [22.38–38.01] $1,000, *p* < 0.001). Table 3 shows the short-term outcomes.

**Table 3:** Postoperative short-term outcomes in study patients.

**Variables**	**Non-PMV (*n* = 85)**	**PMV (*n* = 86)**	***p*-value**
Tracheotomy (n)	0 (0%)	12 (14%)	<0.001
Re-operation for bleeding (n)	1 (1.2%)	5 (5.8%)	0.210
Transfusion (n)	13 (15.3%)	26 (30.2%)	0.028
RRT (n)	0 (0%)	6 (7%)	0.029
ECMO (n)	0 (0%)	4 (4.7%)	0.121
Delirium (n)	5 (5.9%)	15 (17.4%)	0.011
Reperfusion pulmonary edema (n)	1 (1.2%)	8 (9.3%)	0.047
Pulmonary hypertension crisis (n)	0 (0%)	5 (5.8%)	0.059
Pulmonary infection (n)	21 (24.7%)	44 (51.2%)	<0.001
Pericardial effusion (n)	4 (4.7%)	7 (8.1%)	0.563
Arrhythmia (n)	1 (1.2%)	6 (7%)	0.117
ICU LOS (d)	4 (3, 5)	6 (5, 10)	<0.001
Hospital LOS (d)	10 (8, 13)	14.5 (11, 21)	<0.001
Total costs ($1,000)	22.15 (18.59, 25.89)	27.38 (22.38, 38.01)	<0.001
90-day mortality (n)	0 (0%)	3 (3.5%)	0.246

*PMV, prolonged mechanical ventilation; RRT, Renal replacement therapy; ECMO, Extracorporeal membrane oxygenation; ICU, intensive care unit; LOS, length of stay*.

## Discussions

Our study defined PMV as the duration of mechanical ventilation after PEA longer than 48 h, the incidence of which was 50.3% (*n* = 86). Although there was no difference in 90-days mortality between the two groups, all three deaths occurred in the PMV group. PMV was significantly associated with prolonged LOIS, higher rates of postoperative complications, and higher in-hospital medical expenses. Compared to the non-PMV group, the PMV group had an in-hospital costs increase of $5,230. Female sex, prolonged DHCA time, increased postoperative blood product use, elevated postoperative total bilirubin levels, increased preoperative PAP, and elongated postoperative ADRV were independent risk factors for PMV.

PMV has been variously defined in previous studies as exceeding 24 h (28), 48 h (21), 72 h (17), 7 days (18), or 21 days (29) of mechanical ventilation in accordance with previous clinical guidelines, clinical experience or the 90th percentile of the duration of mechanical ventilation for the cohort. We tried to use statistical methods to find the cutoff where the hazard ratio of p-LOHS increased steeply, which was 48 h. The PMV incidence (50.3%) in our study was significantly higher than previous studies for various cardiothoracic surgeries, which may be due to the following reasons. One major reason may be the differences in PMV definitions. Sharma et al. (12) defined PMV as exceeding 48 h. Although their incidence of PMV after cardiac surgery was only 6%, they excluded relatively severe procedures, such as ventricular assist device insertion or heart transplantation. Papathanasiou et al. (18) from Germany identified an incidence of 43% in patients after left ventricular assist device implantation, and their definition of PMV was longer than 7 days. However, the baseline cardiopulmonary dysfunction was relatively severe in our cohort, which directly contributed to postoperative PMV.

The present study used the multiple logistic regression model to identify that female sex, prolonged DHCA time, increased postoperative blood product use, elevated postoperative total bilirubin levels, increased preoperative PAP and elongated postoperative ADRV were independent risk factors for PMV. Notably, several variables that were often considered risk factors for PMV after cardiothoracic surgeries were not identified as risk factors in this study, such as advanced age, increased BMI, low ejection fraction, and emergent surgery (12, 14, 30). The reason may be the characteristics of CTEPH patients. Patients with CTEPH are generally of younger ages, and their BMI distribution is narrow. CTEPH primarily involves the right heart system (1), and the early impact on ejection is relatively small. Moreover, there was no emergent surgery in this study cohort.

The relationship between female sex and mechanical ventilation time after cardiothoracic surgery is controversial (31, 32). Our study found that female sex was a risk factor for PMV after PEA, and female patients had a 2.9-fold increased incidence of PMV. Our findings are supported by the results of many previous studies that showed that female patients had longer mechanical ventilation times and worse outcomes than male patients after cardiac surgeries (28, 33). The exact reason for the sex differences is not known. We hypothesize that it may be related to the hormonal and biological differences between males and females. Butterworth et al. (34) demonstrated that female patients tended to have more opioid exposure after cardiac surgery, which may result in a higher incidence of opioid-induced respiratory depression.

Prolonged DHCA time was the strongest predictor, with a 3.5-fold increased incidence of PMV in our study. Prior studies support this finding (11, 35). Although DHCA provides a bloodless field of vision for the dissection plane and protection for important organs, such as the brain, with the reduction of metabolic demands and oxygen consumption, hypothermia itself has several potential damaging side effects. Previous studies reported that deep hypothermic temperatures may lead to hypothermia-induced coagulation dysfunction, increased systemic inflammatory response, increased capillary permeability, microvasculature endothelial dysfunction, and increased risk of organ dysfunction, such as renal and respiratory failure (36). An increasing number of surgeons recently chose moderate hypothermic circulation in aortic arch surgery because it avoids the adverse effects of hypothermia and decreases CPB times by reducing the cooling and rewarming time (37–39). Prashanth et al. (39) reported that moderate hypothermic circulatory arrest (MHCA) with SACP was safe, with satisfactory neurological outcomes, without increasing mortality and morbidity. Because DHCA time was a modifiable risk factor in this study, we hypothesized that MHCA would be beneficial to the recovery of self-help ventilation compared to DHCA.

The present study found that CPB time was not an independent risk factor for PMV after PEA, which was controversial in previous studies (12, 31). Matthew et al. (11) examined risk factors for prolonged postoperative respiratory support in patients undergoing thoracic aortic surgery and found that CPB time was not associated with the need for prolonged postoperative respiratory support. Maria et al. (18) recently examined predictors of prolonged ventilation in patients who received left ventricular assist device implantation and did not find an association between CPB time and increased risk of PMV. However, the CPB time was a predictor for PMV after cardiac surgery in Sharma et al. (12) and Lei et al. (31), and they believed that prolonged CPB time had adverse effects on pulmonary function by inducing a systemic inflammatory response, producing oxygen free radicals, and activating polymorphonuclear neutrophils. This finding needs further confirmation in future studies.

Similar to previous studies, our study showed that postoperative blood product transfusion was an independent risk factor for PMV. Previous studies found that patients requiring blood product transfusion had increased pulmonary complications, adverse postoperative events, and hospitalization-related costs compared to non-transfusion recipients (11, 40, 41). An analysis of nearly 17,000 patients undergoing CABG surgery showed that the transfusion of even one unit of red blood cells had an adverse impact on the patient prognosis, such as increasing the risk of morbidity and mortality (42). Our findings further emphasized the importance of strengthening blood conservation methods and improving guidelines for blood product transfusion.

In addition, increased preoperative PAP and elongated postoperative RVAD were independent risk factors for postoperative PMV. They contributed to a 1.031- and 1.119-fold increase of postoperative PMV, respectively. The higher the preoperative PH is, the more serious the patient's condition is indicated before the operation, and the longer it takes patients to recover after surgery. Susanna et al. (43) found that preoperative mPAP <16 mmHg is strongly associated with morbidity and mortality after Bidirectional Cavopulmonary Connection, while PVR is not. Patients in the high preoperative mPAP group experienced the longest mechanical ventilation time and p-LOHS, the highest risk of postoperative arrhythmias and need for supplemental oxygen at discharge. Ruchit et al. (44) found that decreasing the patients' mPAP preoperatively can lead to potential benefits for postoperative early surgical outcomes like shortened duration of mechanical ventilation, reduced length of ICU stay and p-LOHS. In turn, elongated postoperative RVAD suggests poorer recovery of right ventricular function after surgery, resulting in patients requiring more recovery time. Several molecular and cellular mechanisms have been proposed in the development of acute RVD secondary to PH (45). RV wall tension increase leads to cardiomyocyte stress and injury secondary to ischemia, substrate depletion, and mitochondrial energy metabolism impairment. Feasible treatment measures may include: (1) General measures include avoiding increased atrioventricular load, reducing atrioventricular contractility and optimizing atrioventricular preload, applying “atrioventricular protective” ventilation strategies to maintain sinus rhythm and atrioventricular synchronization; (2) Medicine therapy, inotropic and vascular activity support (46).

This study is the first study to demonstrate the scientific definition, incidence, and risk factors for PMV in CTEPH patients after PEA. Our findings have multiple implications for perioperative management in CTEPH patients and provide future research directions on pulmonary complications after PEA. Although some risk factors in the present study were not modifiable, including female sex, increased preoperative PAP, elongated postoperative RVAD, and elevated postoperative total bilirubin levels, these parameters may be used to identify patients with a high risk for PMV after PEA. The avoidance or targeted therapy of the modifiable factors, which included decreasing DHCA time and the implementation of a conservative blood transfusion protocol, may help reduce the incidence of PMV, shorten the LOIS and p-LOHS, and reduce unnecessary hospitalization costs. Because MHCA is safe and has satisfactory neurological outcomes, MHCA instead of DHCA may be a better strategy to reduce the risk of PMV in CTEPH patients undergoing PEA.

### Limitations

The present study has several limitations. First, this study is a retrospective, single-institution study involving only PEA, and the application of some conclusions may be limited. With the possibility of missing other important factors that may be related to the mechanical ventilation weaning outcome, such as pre- or post-operative fluid balance, CT and MRI data, the risk model can only be applied within the variables that were included in this study. Second, due to the relatively aggressive definition of PMV in this study, the incidence of PMV and the short-term outcomes of CTEPH patients after PEA may have been overestimated. We have not clarified the underlying etiology of PMV. Third, because of the small sample size, the internal and external validations of the prediction ability were not performed. A large-scale prospective study is needed in the future to derive an exact predictive model of PMV after PEA.

## Conclusions

The incidence (50.3%) of PMV, which was defined using the cutoff value (48 h), after PEA in this study was significantly higher than other surgeries. This finding was likely because of the different definitions of PMV and different degrees of baseline cardiopulmonary dysfunction. Multivariate analysis revealed that female sex, prolonged DHCA time, increased postoperative blood product use, elevated postoperative total bilirubin levels, increased preoperative PAP and elongated postoperative RVAD were independent risk factors for PMV. Compared to the non-PMV group, the PMV group had prolonged LOIS, higher rates of postoperative complications, and higher in-hospital medical expenses. The identification of risk factors associated with PMV in CTEPH patients undergoing PEA may facilitate timely diagnosis and reinterventions for some of these modifiable factors to achieve the goals of decreasing ventilation time and improving patient outcomes.

## Data Availability Statement

The raw data supporting the conclusions of this article will be made available by the authors, without undue reservation.

## Author Contributions

GW and CZ proposed the idea of this investigation. SS, JL, ZF, and LY were responsible for the collection of data and material. CZ helped with the statistical analysis and wrote the manuscript. GW helped to revise the manuscript. All authors contributed extensively to the work presented in this paper, read, and approved the final manuscript.

## Conflict of Interest

The authors declare that the research was conducted in the absence of any commercial or financial relationships that could be construed as a potential conflict of interest.
