# Supplementary material for: Risk Factors for Prolonged Mechanical Ventilation After Pulmonary Endarterectomy: 7 Years' Experience From an Experienced Hospital in China
Source: Front Surg. 2021 Jun 10;8:679273. doi: 10.3389/fsurg.2021.679273 (PMC8222625; doi:10.3389/fsurg.2021.679273)
Supplement: Supplementary file 1 [file Data_Sheet_1.PDF]

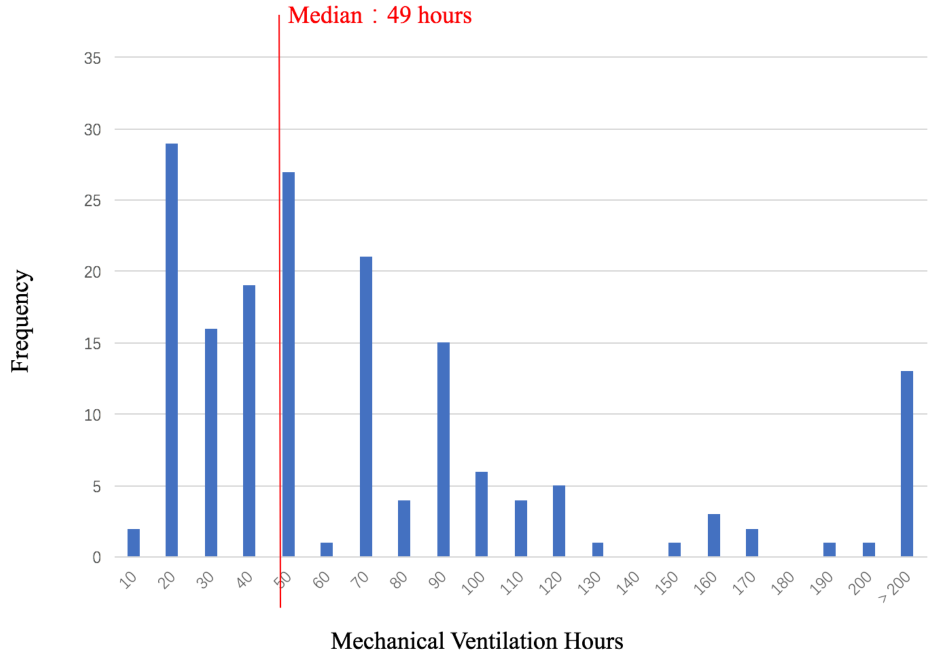


Figure1. the histogram of postoperative mechanical ventilation time

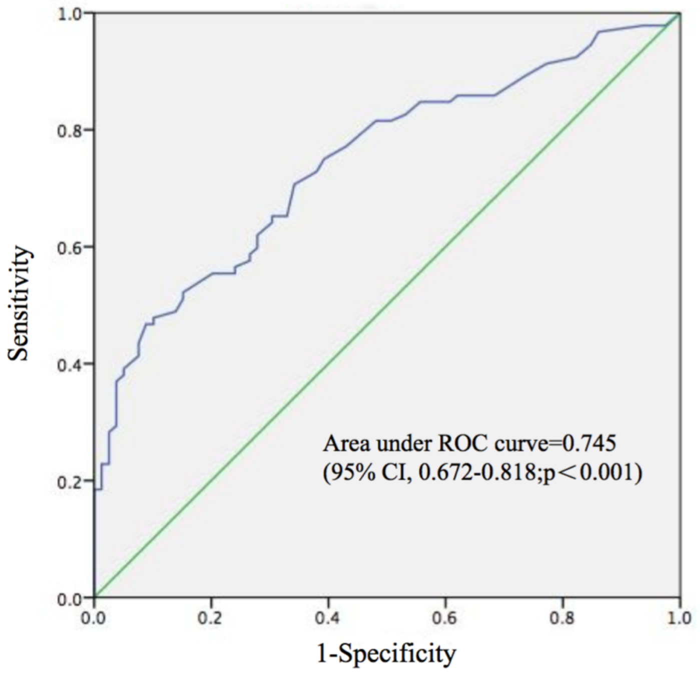


Figure2. The area under the receiver operating characteristic curve (AU-ROC) for the effect of prolonged mechanical ventilation on the prolonged postoperative length of hospital stay. The Youden's index has the maximal value when mechanical ventilation time was around 48 hours.

Table1. Summary of Recent Studies Reporting PMV in Patients After Cardiac Surgery.

| Reference | Patients (n) | Patient Selection | Definition of PMV | Incidence of PMV | Report Independent Risk Factors for PMV |
|---|---|---|---|---|---|
| Vivek et al (6) (2016) | 32 045 | Cardiac surgery | 48 hours | 7% | previous cardiac surgery, lower left ventricular ejection fraction, shock, surgery involving repair of congenital heart disease, and cardiopulmonary bypass time. |
| Angelo et al (7) (2011) | 362 | Complex congenital heart surgery | 7days | 11% | age, greater severity of illness at post- operative admission, healthcare-associated infections, non-infectious pulmonary complications, and the need for re-intervention |
| Maria et al (8) (2019) | 139 | left ventricular assist device implantation | 7days | 43% | previous sternotomy, decreasing e GFR and platelet counts |
| Huan et al (9) (2018) | 382 | Robot-assisted coronary artery bypass graft surgery | 48 hours | 11.3% | age and anesthesia time |
| Maria et al (10) (2018) | 3588 | Cardiac surgery | 24 hours | 11.6% | None |
| Lara et al (11) (2019) | 1994 | Cardiac surgery | 24 hours | 11% | None |
| Qian et al (12) (2009) | 255 | Aortic Arch Surgery | 72 hours | 10% | Prolonged CPB time, Advanced Age, Emergency Creatinine |
| Rajakaruna et (13) al (2005) | 7553 | Cardiac surgery | 96 hours | 2.6% | older age, NYHA class, EF less than 50%, creatinine greater than 200 mol/L, multiple valve replacements, aortic procedures, operative priority, reoperation for bleeding, inotropes, and preoperative IABP |
| Hesham et al (14) (2012) | 10 977 | Elective coronary surgery | 72 hours | 1.96% | NYHA higher than class II, renal dialysis, age, reduced $FEV_1$, BMI >35 $kg/m^2$ |
| Joanne et al (15) (2019) | 1257 | Cardiac surgery | 48 hours | 15.9% | diabetes mellitus, preoperative RRT, need for intraoperative transfusion, combined coronary bypass/valular surgery and intra-aortic balloon pump. |
